# Respiratory chronic health conditions and racial disparities associated with e-cigarette use: a cross-sectional analysis using behavioral risk factor surveillance data

**DOI:** 10.3389/fpubh.2024.1497745

**Published:** 2024-12-10

**Authors:** Ashley Comiford, Steven Pan, Sixia Chen

**Affiliations:** ^1^Cherokee Nation Health Services, Tahlequah, OK, United States; ^2^Department of Biostatistics and Epidemiology, Hudson College of Public Health, University of Oklahoma Health Sciences, Oklahoma City, OK, United States

**Keywords:** COPD, asthma, electronic cigarettes, cigarettes, health disparities

## Abstract

**Background:**

Chronic Obstructive Pulmonary Disease (COPD), mainly caused by cigarette smoking, is one of the leading causes of death in the United States (US) and frequent asthma attacks are often exacerbated by cigarette use. Electronic cigarettes (e-cigarettes) are often used to quit cigarette smoking. Prevalence of COPD, asthma, cigarette use, and e-cigarette use differs between racial/ethnic groups. The overall objective was to assess the associations between e-cigarette use and COPD and asthma and how race/ethnicity and cigarette smoking modifies these associations.

**Methods:**

Data were retrieved from the 2016–2018 and 2020–2021 Behavioral Risk Factor Surveillance System datasets, a national annual health survey representing the US general adult population. Frequency and weighted percentages or means and standard deviations were obtained. Rao-Scott Chi-square test, two-sample *t* tests, and logistic regression were used to evaluate binary associations between current e-cigarette use and lifetime diagnosis of COPD and asthma. Multivariable analyses using logistic regression were conducted to assess associations between variables. Interaction effects between e-cigarette use and race/ethnicity were assessed and stratified analyses were performed as indicated. All multivariate analyses were stratified by cigarette smoking status.

**Results:**

Prevalence of e-cigarette use was 5.1%, COPD was 6.7%, and asthma was 9.2%. Individuals who currently smoked cigarettes among all racial/ethnic groups, excluding non-Hispanic (NH) American Indian/Alaska Native individuals, were more likely to report current asthma if using e-cigarettes compared to non-use (*p* < 0.05). Among individuals who never smoked, Non-Hispanic White (NHW), NH-Black and Hispanic individuals using e-cigarettes had greater odds of COPD compared to NHW, NH-Black and Hispanic individuals who did not use these products, respectively (*p* < 0.05). Among NHW, Hispanic, and NH-Other persons who currently used cigarettes, individuals currently using e-cigarettes had greater odds of COPD compared to NHW, Hispanic, and NH-Hispanic individuals who did not use e-cigarettes, respectively (*p* < 0.05). Among individuals who formerly used cigarettes, current e-cigarette use was associated with COPD and asthma. Among individuals who never used cigarettes, current e-cigarette use was associated with reporting current asthma.

**Conclusion:**

The association between e-cigarette use and COPD and asthma was dependent on smoking status and racial/ethnic groups. Further studies should be conducted to explore this association.

## Introduction

With more than sixteen million adults in the United States (US) diagnosed with Chronic Obstructive Pulmonary Disease (COPD) (1), it is one of the leading causes of death (2). COPD refers to respiratory conditions, including emphysema and chronic bronchitis, that result in continuous lung function decline and respiratory failure (3). Asthma, another lung condition, can cause repeated episodes of wheezing breathlessness, chest tightness, and coughing, and approximately 8% of US adults have asthma (4).

With both COPD and asthma, there are racial/ethnic disparities. For example, 2017 Behavioral Risk Factor Surveillance System (BRFSS) data indicated that 11.9% of American Indian/Alaska Native (AI/AN) adults reported having COPD compared to 6.7% of non-Hispanic White (NHW) adults, 6.6% of Black adults, 3.6% of Hispanic adults, and 1.7% of Asian adults (5). According to 2021 National Health Interview Survey (NHIS) data, about 13% of AI/AN adults reported having asthma compared to 8% of NHW adults, 10.7% of Black adults, 6.7% of Hispanic adults, and 4.2% of Asian adults (4).

Cigarette smoking is the leading cause of COPD and is associated with 80% of COPD-related deaths (6). Approximately 21% of US adults with asthma smoke cigarettes despite cigarette smoke intensifying asthma symptoms (7). Comparatively, only 16.8% of US adults without asthma smoke cigarettes (7). As with COPD and asthma, there are racial/ethnic disparities in the prevalence of cigarette smoking. Analysis of 2020 NHIS data indicated that 27.1% of AI/AN adults reported currently smoking cigarettes compared to 13.3% of NHW adults, 14.4% of Black adults, 8.0% of Hispanic adults, and 8.0% of Asian adults (8).

Electronic cigarettes (e-cigarettes) have become popular over the past decade to quit or reduce cigarette smoking (9). In 2021, 4.5% of US adults aged 18 years and older currently used e-cigarettes, and e-cigarette use varied by race/ethnicity (10). The prevalence of e-cigarette use among NHW adults (5.2%) was higher than that of Asian adults (2.9%), Black adults (2.4%), and Hispanic (3.3%) adults (9). Data from the 2014 NHIS showed that ever use of e-cigarettes was higher among the AI/AN population (20.2%) compared to the NHW population (14.8%) (11). Current use was also higher among the AI/AN population (10.7%) than the NHW population (4.6%) (11).

Individuals with COPD and/or asthma may be more motivated to use e-cigarettes to reduce the known poor respiratory health outcomes related to sustained cigarette smoking after diagnosis (12, 13). As such, several studies have found an association between e-cigarette use and COPD and/or asthma (14–18). Yet, another study indicated that dual use of e-cigarettes and cigarettes was associated with worse health-related quality of life compared to exclusive cigarette use (19). Concerns also remain regarding the implications of e-cigarette use on COPD and asthma outcomes, including increased risk of decline in lung health or exacerbations given the irritants and chemicals in e-cigarettes (18–20). Given the racial/ethnic disparities in cigarette use, e-cigarette use, and the prevalence of COPD and asthma, it is important to explore how race/ethnicity affects this association.

The first objective of this study was to assess the prevalence of e-cigarette use by race/ethnicity using a nationally representative dataset. We also aimed to assess the association between e-cigarette use and COPD and asthma and how race/ethnicity and cigarette smoking may modify these associations. We hypothesize that e-cigarette use will be associated with both COPD and asthma across race/ethnicity groupings while controlling for smoking status.

## Materials and methods

### Population

The 2016–2018 and 2020–2021 BRFSS datasets were used in this analysis. The 2019 BRFSS dataset was not used as it did not contain all variables of interest, particularly e-cigarette use. The BRFSS is a US surveillance system in which state-level data on various health risk behaviors, preventive health practices, and health care access primarily related to chronic disease and injury is collected. The BRFSS primarily uses telephone surveys to collect data. It involves random-digit-dialing to select a representative sample of non-institutionalized adults (18 years and older) in each state and certain territories. It employs a complex weighting procedure to ensure that the data collected is representative of the adult population within each state. The main components of the weighting procedure include the calculation of base weight, Post-Stratification, Non-Response Adjustment, Weight Trimming, and Calibration (21). For more details about sampling and weighting of BRFSS, see https://www.cdc.gov/brfss/index.html.

### Measures

#### Outcome variables

The two main outcome variables were COPD and asthma and were defined as ever being told one had COPD or asthma. In the BRFSS survey, participants were asked the following question, “Has a doctor, nurse, or other health professional ever told you that you had C.O.P.D. (chronic obstructive pulmonary disease), emphysema or chronic bronchitis? COPD was categorized as yes vs. no. For asthma, participants were asked, “Has a doctor, nurse, or other health professional ever told you that you had asthma?” and “Do you still have asthma?” If participants answered not and yes to both questions, the asthma variable was categorized as current. If they answered yes to the first question but no to the second question, the asthma variable was categorized as former. If they answered no to both questions, the variable was categorized as never.

#### Independent variable

The main independent variable was current e-cigarette use. This variable was based on the following question, “Do you now use e-cigarettes or other electronic “vaping” products every day, some days, or not at all?” Adults who used e-cigarettes every day or some days were identified as currently using e-cigarettes versus not using e-cigarettes at all (yes/no).

#### Stratification variables

Race/ethnicity was categorized as NHW (reference), Non-Hispanic (NH) Black, NH-AI/AN, Hispanic, and NH-other. The race/ethnicity variable was only stratified in the analyses if the interaction effect of race/ethnicity and current e-cigarette use was significant. Smoking status was categorized as current, former, never (reference). Smoking status was based on two questions, “Have you smoked at least 100 cigarettes in your entire life?” and “Do you now smoke cigarettes every day, some days, or not at all?” Current use was defined as answering yes to the first question and every day or some days to the second question. Former use was defined as answering yes to the first question and not at all to the second question. Never use was defined as answering no to the first question.

#### Other covariate variables

We included the several covariates to control for confounding based on existing literature and previous resource experience (22–24). The following variables were selected and references are in bold: sex (**male**/female), age group (**18–24**, 25–34, 35–44, 45–54, 55–64, 65 and older), region (**Northeast**, Midwest, South, West), education (**Less than high school** and high school or higher), income (**<$10,000**, $10,000–$15,000, $15,000–$20,000, $20,000–$25,000, $25,000–$35,000, $35,000–$50,000, $50,000–$75,000, $75,000 or higher), employment (yes/**no**), marital status (yes/**no**), number of children in the household (mean ± standard deviation (SD), general health status (**excellent**/very good/good/fair/poor), number of days where physical health was not good (mean ± SD), number of days where mental health was not good (mean ± SD), Body Mass Index (BMI) (mean +/- SD), having health care coverage (yes/**no**), access to health care professionals (yes/**no**), length of time since last routine checkup (within past year/within past 2 years/within past 5 years/5 or more years ago/**never**), exercise in past 30 days (yes/**no**), smoking status (current/former/**never**), smokeless tobacco use (every day/some days/**not at all**), alcohol use in past 30 days (yes/**no**). Sex, age group, region, education, income, employment, marital status, physical and mental health days, BMI, insurance coverage, access to health care, time since last checkup, exercise, smokeless tobacco status, and alcohol use were based on current status. Education was dichotomized due to small cell sizes for the AI/AN stratum. We dichotomized education based on previous literature (25). For the rest of the categorical variables, all variables included the levels available in the dataset. For more detailed information about the BRFSS questionnaire, see https://www.cdc.gov/brfss/index.html.

### Statistical methods

In this study, we aggregated the data from the 2016–2018 and 2020–2021 BRFSS files. A novel stratification variable was created by incorporating the year of the survey along with the original stratification variable present in each file. To account for the combined multi-year dataset, a composite weight was computed for each observation, derived by dividing the original weight by the number of years (e.g., 5). Descriptive statistics were computed for continuous variables, including the weighted mean and weighted SD. For categorical variables, descriptive statistics encompassing the weighted frequency and weighted percentages were calculated. To assess disparities in covariate distributions between current e-cigarette use and non-current e-cigarette use, Rao-Scott Chi-square tests were employed for categorical variables, and weighted two-sample *t* tests were utilized for continuous variables. Binary associations between each covariate variable and outcome variables were investigated through weighted logistic regression models. Additionally, multivariate associations between the outcome variables and both independent and covariate variables were examined using weighted logistic regression models using backward selection to select the final model. To ensure the robustness of the findings, collinearity was assessed. Interaction effects between racial/ethnic and e-cigarette use status were evaluated within each smoking stratum. Stratified analyses were performed as indicated. Missing values were systematically excluded from the analysis to uphold the integrity of the results. All statistical analyses were conducted in SAS 9.4.

## Results

Table 1 presents the weighted characteristics of the study population. In this population, 51.7% were female, 12.3% were aged 18 to 24 years, 16.9% were aged 25 to 34 years, 16.2% were aged 35 to 44 years, 16.2% were aged 45 to 54 years, 16.9% were aged 55 to 64 years, and 21.5% were 65 years or older. Most of the population were NHW (63.4%), 11.8% were NH-Black, 16.6% were Hispanic, 0.9% were NH-AI/AN, and 7.2% were some other racial/ethnic group. The majority of the population had a high school diploma or higher (87.0%). More than 35.8% of the population reported an income of $75,000 or higher. The majority of the population reported being employed (56.8%) and half reported being married (50.6%). Nearly 83% of the population reported good to excellent health with the mean number of days health was not good being 3.81 (SD ±0.013). Close to 90% reported having health coverage, approximately 80% indicated they had access to health care professionals, and more than 70% have had a routine health checkup within the past year.

**Table 1:** Overall weighted descriptive summary and by current e-cigarette use.

Variables	Current e-cigarette use *n* (%)*	Non-current e-cigarette use *n* (%)*	Overall *n* (%)*	*p*-value
Current e-cigarette use	NA	NA	66,965 (5.1%)	NA
Sex, Female	30,975 (41.2%)	965,564 (52.2%)	996,539 (51.7%)	<0.0001
Age group				<0.0001
18–24	14,282 (30.8%)	89,628 (11.3%)	103,910 (12.3%)	
25–34	14,975 (26.9%)	170,330 (16.4%)	185,305 (16.9%)	
35–44	11,351 (16.9%)	205,581 (16.1%)	216,932 (16.2%)	
45–54	10,129 (11.9%)	266,973 (16.5%)	277,102 (16.2%)	
55–64	9,859 (9.1%)	364,984 (17.3%)	374,843 (16.9%)	
65 and older	6,369 (4.3%)	633,767 (22.4%)	640,136 (21.5%)	
Race				<0.0001
NH-White	50,949 (70%)	1,332,636 (63.1%)	1,383,585 (63.4%)	
NH-Black	3,734 (8.6%)	136,452 (12%)	140,186 (11.8%)	
NH-AI/AN	1,443 (1.3%)	28,815 (0.9%)	30,258 (0.9%)	
Hispanic	5,498 (12.7%)	139,312 (16.8%)	144,810 (16.6%)	
NH-Other	5,341 (7.3%)	94,048 (7.2%)	99,389 (7.2%)	
Smoking status				<0.0001
Current use	30,386 (42%)	225,573 (13.9%)	255,959 (15.3%)	
Former use	22,953 (32.4%)	482,204 (23.7%)	505,157 (24.1%)	
Never use	13,626 (25.6%)	1,023,486 (62.4%)	1,037,112 (60.6%)	
Region				<0.0001
Northeast	12,267 (15.7%)	344,646 (17.1%)	356,913 (17.1%)	
Midwest	18,131 (22.6%)	478,698 (21.3%)	496,829 (21.4%)	
South	20,897 (41.2%)	532,115 (39.5%)	553,012 (39.6%)	
West	15,670 (20.5%)	375,804 (22.1%)	391,474 (22%)	
Education				0.882
Less than high school	5,656 (12.9%)	118,339 (13%)	123,995 (13%)	
High school or higher	61,189 (87.1%)	1,608,680 (87%)	1,669,869 (87%)	
Income				<0.0001
< $10,000	3,498 (5.7%)	59,918 (5.2%)	63,416 (5.2%)	
< $15,000	3,421 (4.8%)	65,939 (4.6%)	69,360 (4.6%)	
< $20,000	4,748 (7.6%)	94,124 (6.8%)	98,872 (6.8%)	
< $25,000	5,947 (9.9%)	120,250 (8.4%)	126,197 (8.4%)	
< $35,000	7,379 (12.5%)	158,162 (10.6%)	165,541 (10.7%)	
< $50,000	8,580 (14.5%)	203,871 (13.1%)	212,451 (13.1%)	
< $75,000	8,922 (15.8%)	238,913 (15.2%)	247,835 (15.2%)	
$75,000 or higher	14,838 (29.2%)	508,947 (36.2%)	523,785 (35.8%)	
BMI (mean (SD))	27.56 (0.006)	28.21 (0.001)	28.18 (0.0112)	<0.0001
Employed	39,973 (63.9%)	861,283 (56.4%)	901,256 (56.8%)	<0.0001
Married	21,524 (30%)	916,088 (51.7%)	937,612 (50.6%)	<0.0001
Number of children (mean (SD))	0.73 (0.009)	0.69 (0.002)	0.69 (0.002)	<0.0001
General health status				<0.0001
Excellent	9,153 (15.8%)	301,244 (18.7%)	310,397 (18.6%)	
Very good	20,065 (31.3%)	579,800 (32.3%)	599,865 (32.3%)	
Good	22,246 (33%)	538,359 (31.6%)	560,605 (31.7%)	
Fair	10,841 (14.7%)	225,387 (13%)	236,228 (13.1%)	
Poor	4,496 (5.1%)	82,578 (4.4%)	87,074 (4.4%)	
Number of days where physical health was not good (mean (SD))	4.69 (0.036)	3.76 (0.006)	3.81 (0.013)	<0.0001
Number of days where mental health was not good (mean (SD))	8.01 (0.039)	3.93 (0.006)	4.14 (0.014)	<0.0001
Having health care coverage	56,694 (84.3%)	1,593,676 (89.1%)	1,650,370 (88.8%)	<0.0001
Access to health care professionals				<0.0001
Yes, only one	40,958 (57.9%)	1,260,565 (68.4%)	1,301,523 (67.9%)	
More than one	7,755 (11.4%)	211,681 (11.5%)	219,436 (11.5%)	
No	17,803 (30.7%)	250,575 (20.1%)	268,378 (20.6%)	
Smokeless tobacco use				<0.0001
Every day	1769 (2.6%)	32,604 (1.8%)	34,373 (1.9%)	
Some days	3,218 (5.5%)	22,412 (1.4%)	25,630 (1.6%)	
Not at all	61,901 (91.9%)	1,674,617 (96.7%)	1,736,518 (96.5%)	
Exercise in past 30 days	48,973 (76.5%)	1,279,847 (75%)	1,328,820 (75.1%)	<0.0001
Alcohol in past 30 days	40,696 (65.1%)	874,251 (52%)	914,947 (52.7%)	<0.0001
Length of time since last routine checkup				<0.0001
Within past year	43,054 (62.9%)	1,330,002 (73.8%)	1,373,056 (73.2%)	
Within past 2 years	9,264 (15.1%)	187,194 (12.6%)	196,458 (12.7%)	
Within past 5 years	6,480 (10.7%)	95,522 (6.9%)	102,002 (7.1%)	
5 or more years ago	6,387 (10.2%)	86,768 (5.8%)	93,155 (6%)	
Never	606 (1.1%)	11,443 (0.9%)	12,049 (0.9%)	
COPD	8,519 (9%)	140,291 (6.6%)	148,810 (6.7%)	<0.0001
Asthma status				<0.0001
Current	8,570 (12.2%)	162,129 (9.1%)	170,699 (9.2%)	
Former	4,294 (7.5%)	66,206 (4.5%)	70,500 (4.6%)	
Never	53,346 (80.4%)	1,491,485 (86.5%)	1,544,831 (86.1%)	

*unweighted frequency (weighted percent).

Only 5.1% of respondents reported current use of e-cigarettes. However, 15.3% of the study population reported currently using cigarettes every day or some days. Approximately 6.7% of the population indicated a diagnosis of COPD and 9.2% reported current asthma.

Table 1 also presents the characteristics of the population by e-cigarette use status. All variables excluding education were significantly associated with e-cigarette use. For example, 41.2% of individuals reporting current e-cigarette use were female compared to more than 52.2% of persons not currently using e-cigarette. E-cigarette use status also differed based on race/ethnicity status. Among study participants currently using e-cigarettes, 70% were NHW, 8.6% were NH-Black, 1.3% were NH-AI/AN, and 12.7% were Hispanic. Comparatively, among individuals not using e-cigarettes, 63.1% were NHW, 12.0% were NH-Black, 0.9% were NH-AI/AN, and 16.8% were Hispanic. Overall health also varied among participants reporting current e-cigarette use compared to those who did not use e-cigarettes. Among individuals who did not use e-cigarettes, only 13.9% reported current cigarette use, and 23.7% former cigarette use. However, persons currently using e-cigarettes overwhelmingly reported either current (42%), or former cigarette use (32.4%). Finally, reviewing chronic diseases, individuals who used e-cigarettes currently were more likely to report having COPD (9.0%) and, former (7.5%) or current asthma (12.2%) compared to those who did not use e-cigarettes (6.6, and 4.5%, 9.1%, respectively).

Table 2 presents the unadjusted weighted binary associations between e-cigarette use and COPD and asthma status. Additionally, this table presents unadjusted associations between cigarette smoking status and race with COPD and asthma. Compared to individuals who did not use e-cigarettes, participants who currently used e-cigarettes had greater odds of COPD (Odds Ratio (OR) = 1.40; 95% Confidence Interval (95% CI) = 1.34–1.47) and asthma (OR = 1.44; 95% CI = 1.38–1.51). As expected, individuals who used cigarettes currently and formerly had greater odds of COPD and asthma when compared to individuals who never used cigarettes. When assessing the association between race/ethnicity and COPD, NH-AI/AN individuals had greater odds (OR = 1.56; 95% CI = 1.44–1.69) when compared to NHW individuals. However, NH-Black individuals (OR = 0.83; 95% CI = 0.80–0.86), Hispanic adults (OR = 0.45; 95% CI = 0.42–0.47), and NH-Other adults (OR = 0.45; 95% CI = 0.42–0.48) had lower odds of reporting COPD when compared to NHW adults. For asthma, both NH-Black individuals (OR = 1.25; 95% CI = 1.21–1.30) and NH-AI/AN individuals (OR = 1.66; 95% CI = 1.53–1.804) had greater odds of reporting current asthma compared to NHW individuals. Yet, compared to NHW adults, Hispanic adults (OR = 0.77; 95% CI = 0.74–0.81) and NH-other adults (OR = 0.75; 95% CI = 0.71–0.79).

**Table 2:** Unadjusted associations between current e-cigarette use, smoking status, and race/ethnicity and COPD and asthma status.

	COPD	Asthma (Current vs. Never)
	OR (95% CI)	
Current e-cigarette use	1.4 (1.34, 1.47)	1.44 (1.38, 1.51)
Smoking status		
Current – every day/some days	6.22 (6.02, 6.42)	1.49 (1.45, 1.54)
Former	4.05 (3.92, 4.17)	1.15 (1.12, 1.18)
Never	1.0 (ref)	
Race/Ethnicity		
NHW	1.0 (ref)	
NH-Black	0.83 (0.8, 0.86)	1.25 (1.21, 1.3)
NH-AI/AN	1.56 (1.44, 1.69)	1.66 (1.53, 1.8)
Hispanic	0.45 (0.42, 0.47)	0.77 (0.74, 0.81)
NH-Other	0.45 (0.42, 0.48)	0.75 (0.71, 0.79)

Table 3 presents the 15 multivariate logistic regression models assessing the association between e-cigarette use and COPD, as well as asthma stratified by smoking status and racial/ethnic group. Table 3 only shows the variables of interest; however, Supplementary Tables S1, S3, S4 present results from the full models for each analysis.

**Table 3:** Multivariate regression models between current e-cigarette use and COPD and asthma, stratified by race/ethnicity and smoking status, adjusted for significantly associated covariates.

	COPD among individuals currently using cigarettes	COPD among individuals never using cigarettes	Asthma (Current vs. Never) among individuals currently using cigarettes
	OR (95% CI)		
NHW, current e-cigarette use	1.1 (1.01, 1.2)	1.49 (1.13, 1.95)	1.2 (1.09, 1.3)
NH-Black, current e-cigarette use	1.19 (0.9, 1.58)	2.01 (1.07, 3.78)	1.48 (1.13, 1.93)
NH-AI/AN, current e-cigarette use	1.31 (0.85, 2)	3.23 (0.53, 19.83)	0.96 (0.6, 1.54)
Hispanic, current e-cigarette use	1.49 (1.06, 2.09)	1.76 (1.08, 2.87)	1.97 (1.45, 2.67)
NH-Other, current e-cigarette use	1.04 (0.74, 1.46)	0.66 (0.29, 1.52)	1.5 (1.03, 2.18)

### NHW individuals

According to Table 3, overall, among NHW persons currently using or never using cigarettes, e-cigarette use was significantly associated with COPD (currently using cigarettes: adjusted OR (aOR) = 1.10, 95% CI = 1.01, 1.20; never using cigarettes: aOR = 1.49, 95% CI = 1.13, 1.95). In addition, among NHW individuals who reported cigarette use, e-cigarette use was also significantly associated with having current asthma (aOR = 1.21; 95% CI = 1.09, 1.30).

### NH-black individuals

According to Table 3, among NH-Black participants who never used cigarettes, persons who reported current e-cigarette use had greater odds of reporting COPD compared to those not reporting e-cigarette use (aOR = 2.02; 95% CI = 1.08, 3.78). Among NH-Black participants who currently used cigarettes, current e-cigarette use was significantly associated with reporting current asthma (aOR = 1.48; 95% CI = 1.13, 1.93).

### NH-AI/AN individuals

According to Table 3, among NH-AI/AN individuals, there were no significant associations between e-cigarette use and COPD or asthma among individuals currently or never using cigarettes.

### Hispanic individuals

According to Table 3, among Hispanic individuals who currently or never used cigarettes, persons reporting current e-cigarette use had greater odds of reporting COPD than those who did not use e-cigarettes (among individuals currently using of cigarettes: aOR = 1.49; 95% CI = 1.06, 2.09) and (among individuals never using cigarettes: aOR = 1.76; 95% CI = 1.08, 2.87). Additionally, among Hispanic individuals who were current users of cigarettes, e-cigarette use was also associated with reporting current asthma (aOR = 1.97; 95% CI = 1.45, 2.67).

### NH-other individuals

According to Table 3, among NH-other individuals who never smoked, individuals reporting current e-cigarette use had significantly higher odds of reporting current asthma (aOR = 1.50; 95% CI = 1.03, 2.18).

When evaluating current e-cigarette use with COPD among former smokers (Table 4), we found that individuals reporting current e-cigarette use had greater odds of reporting COPD (aOR = 1.27; 95% CI = 1.13, 1.42) when controlling for race/ethnicity and other variables. When assessing the association between e-cigarettes with asthma, among never users of cigarettes, individuals reporting current e-cigarette use had greater odds of reporting current asthma compared to those not reporting e-cigarette use (aOR = 1.17; 95% CI = 1.02, 1.34) (Table 4). Among participants who formerly smoked, current e-cigarette use was not significantly associated with reporting current asthma. Supplementary Tables S2, S5 present results from the full models shown in Table 4.

**Table 4:** Multivariate regression analyses between current e-cigarette use and COPD and asthma, stratified by smoking status, adjusted for significantly associated covariates.

	COPD	Asthma	
		Current vs. Never	Current vs. Never
	Among individuals formerly using cigarettes		Among individuals never using cigarettes
	OR (95% CI)		
Current e-cigarette use	1.27 (1.13, 1.42)	0.95 (0.86, 1.05)	1.17 (1.02, 1.34)
Race			
NH-White	1.0 (ref)		
NH-Black	0.67 (0.6, 0.74)	1.06 (0.97, 1.17)	1.03 (0.97, 1.09)
NH-AI/AN	1.04 (0.88, 1.22)	1.34 (1.13, 1.58)	1.17 (1.02, 1.35)
Hispanic	0.43 (0.38, 0.49)	0.79 (0.71, 0.88)	0.71 (0.66, 0.76)
NH-Other	0.74 (0.62, 0.89)	1.05 (0.92, 1.19)	0.69 (0.64, 0.76)

## Discussion

In our study, we found that individuals who currently used cigarettes, among all racial/ethnic groups, excluding NH-AI/AN individuals, were more likely to report current asthma if they were also currently used e-cigarettes compared to individuals who did not use e-cigarettes. Among individuals who never used cigarettes, NHW, NH-Black, and Hispanic adult e-cigarette users had greater odds of reporting COPD compared to their non-e-cigarette users counterparts. Among individuals currently smoking cigarettes, NHW and Hispanic individuals using e-cigarettes had greater odds of reporting COPD compared to their respective NHW and Hispanic non-users. Among individuals who formerly used cigarettes, current e-cigarette use was associated with COPD and asthma. Among individuals who never used cigarettes, current e-cigarette use was associated with reporting current asthma.

The results of our study are similar to what previous studies have found. For example, a study conducted by Bircan et al. analyzing 2016–2018 BRFSS data found that e-cigarette use, among never smokers, had increased odds of COPD and asthma compared to never users (14). Another study also utilizing BRSS data found similar results (15). In another study using the Population Assessment of Tobacco and Health (PATH) longitudinal data, when compared to never tobacco use, current exclusive cigarette use and dual use, use of cigarettes and e-cigarettes concurrently were associated with increased COPD prevalence (17). However, COPD prevalence did not differ between exclusive e-cigarette use and exclusive cigarette use (17). That study found that individuals using e-cigarette or cigarette exclusively had similar cigarette smoking backgrounds; emphasizing that cigarette use may be driving the association between e-cigarette use and COPD (17). Another study using PATH longitudinal data found no association between e-cigarette use and COPD after controlling for current cigarette smoking and cigarette pack years (16). Moreover, another study using National Health and Nutrition Examination Survey data indicated that prevalence of COPD and asthma were more prevalent among individuals who used both products when compared to exclusive use of cigarettes and e-cigarettes (26).

To our knowledge, our study is the first to assess how race/ethnicity affects the association between e-cigarettes and COPD and asthma. While other studies have adjusted for race/ethnicity when assessing the relationship between e-cigarettes and COPD/asthma (14–17), our study took it a step further and analyzed the association of e-cigarettes and COPD/asthma by ethnicity/racial groupings. Since e-cigarettes can cause acute health issues, including damage to the lungs (18, 20), there may be concern about the short and long-term impact of e-cigarette use among individuals already experiencing lung issues including those diagnosed with COPD and asthma. Thus, the potential health benefits of switching to e-cigarettes among this population may be reduced compared to the smoking cessation benefits e-cigarettes may have in the general population (27). This may have implications for clinical care of individuals who smoke cigarettes and use e-cigarettes. Clinicians and public health practitioners may want to inquire about e-cigarette use among individuals with COPD and/or asthma and encourage those individuals to use evidence-based smoking methods to quit the use of cigarettes. Clinicians may also want to inquire about e-cigarette use among individuals who never used cigarettes as individuals who never used cigarettes but currently use e-cigarettes had greater odds, among certain racial/ethnic groups, of reporting COPD. Interventions to prevent and reduce e-cigarette use is warranted until long-term studies can be done to assess the potential risk of e-cigarette use and the development of COPD or asthma. Further research should be done to identify the temporal relationship between the association of e-cigarette use and COPD and asthma and the effects of e-cigarettes on the health of individuals diagnosed with COPD and/or asthma.

Our study has some limitations. This study is only observational and cannot be used for obtaining a causal relationship between the use of e-cigarettes and COPD and asthma. The current study is cross-sectional, and a longitudinal study might provide more comprehensive information. Other potential confounding variables including genetic information, allergy status, environmental factors (quality of air and exposure to second-hand smoking and chemicals), nutrition intake, and the intensity of using different kinds of tobacco products were not available in our data file. This data is also self-reported and as such, the prevalence of the independent and dependent variables of interest may be underreported due to social desirability and stigma-related concerns. This may bias the results toward the null. Despite these limitations, this is a large nationally represented study with a sample size large enough to analyze by racial/ethnic groups. Particularly, we were able to analyze AI/AN as its own racial/ethnic group that is often grouped in the “other” category.

## Conclusion

In conclusion, in this study, the association between e-cigarette use and COPD and asthma was dependent on smoking status and racial/ethnic grouping. Clinicians and public health official should encourage individuals with COPD and asthma to use evidence-based methods for cigarette smoking cessation and caution them about the potential risk of e-cigarettes. Additionally, those who have never smoked should be cautioned about e-cigarette use until further long-term studies can be done on the effects of e-cigarettes. Further studies, including longitudinal studies, should be conducted to determine the directionality of this association. It is especially important to study these associations among vulnerable populations that already experience disparities in smoking and smoking-related morbidity and mortalities.

## Data Availability

Publicly available datasets were analyzed in this study. This data can be found here: https://www.cdc.gov/brfss/annual_data/annual_data.htm.
