# Supplementary material for: Respiratory chronic health conditions and racial disparities associated with e-cigarette use: a cross-sectional analysis using behavioral risk factor surveillance data
Source: Front Public Health. 2024 Dec 10;12:1497745. doi: 10.3389/fpubh.2024.1497745 (PMC11666483; doi:10.3389/fpubh.2024.1497745)
Supplement: Supplementary file 1 [file Table_1.DOCX]

**Supplemental**

Table S1. Multivariate Weighted Regression Analyses for Chronic Obstructive Pulmonary Disease among Current Smokers, Stratified by Race, BRFSS 2016-2018, 2020-21 Combined, Adjusted for Significantly Associated Covariates

|  | COPD | | | | |
| --- | --- | --- | --- | --- | --- |
|  | NH-White | NH-Black | NH-AI/AN | Hispanic | NH-Other |
|  | OR (95% CI) | | | | |
| Current E-Cigarette Use | 1.1 (1.01, 1.2) | 1.19 (0.9, 1.58) | 1.31 (0.85, 2) | 1.49 (1.06, 2.09) | 1.04 (0.74, 1.46) |
| Sex, Female | 1.26 (1.19, 1.34) | 1.58 (1.33, 1.88) | 1.58 (1.17, 2.14) | 1.46 (1.08, 1.97) | 1.44 (1.09, 1.9) |
| Age Group |  | | | | |
| 18-24 | 1.0 (ref) | | | | |
| 25-34 | 1.25 (0.99, 1.58) | 1.65 (0.84, 3.24) | 0.47 (0.15, 1.52) | 1.31 (0.78, 2.21) | 1.42 (0.78, 2.57) |
| 35-44 | 2.02 (1.62, 2.52) | 1.59 (0.82, 3.07) | 1.31 (0.45, 3.76) | 1.69 (1, 2.88) | 1.91 (1.06, 3.46) |
| 45-54 | 3.52 (2.83, 4.37) | 2.12 (1.11, 4.05) | 1.79 (0.67, 4.79) | 1.88 (1.12, 3.16) | 2.7 (1.46, 5.01) |
| 55-64 | 5.04 (4.06, 6.24) | 3.41 (1.79, 6.51) | 1.91 (0.71, 5.14) | 4.13 (2.15, 7.92) | 4.41 (2.44, 7.98) |
| 65 and older | 6.9 (5.54, 8.6) | 3.69 (1.9, 7.18) | 3 (1.09, 8.27) | 3.95 (2.17, 7.17) | 9.04 (4.63, 17.69) |
| Region |  | | | | |
| Northeast | 1.0 (ref) | | | | |
| Midwest | 1 (0.93, 1.09) | 1.42 (1.11, 1.81) | 0.9 (0.52, 1.55) | 1.15 (0.79, 1.69) | 1.38 (0.94, 2.01) |
| South | 1.14 (1.05, 1.24) | 1.37 (1.09, 1.73) | 1.09 (0.63, 1.9) | 0.9 (0.64, 1.27) | 1.4 (0.95, 2.05) |
| West | 0.9 (0.81, 0.99) | 0.83 (0.56, 1.23) | 0.43 (0.24, 0.76) | 0.74 (0.53, 1.02) | 0.88 (0.56, 1.38) |
| Education |  | | | | |
| Less than high school | 1.0 (ref) | | | | |
| High school or more | 0.73 (0.67, 0.79) | 0.74 (0.61, 0.91) | 1.03 (0.71, 1.48) | 0.77 (0.58, 1.04) | 0.63 (0.44, 0.9) |
| Income |  | | | | |
| < $10,000 | 1.0 (ref) | | | | |
| < $15,000 | 1.07 (0.95, 1.21) | 0.87 (0.65, 1.15) | 0.99 (0.61, 1.6) | 1.34 (0.84, 2.11) | 0.96 (0.62, 1.48) |
| < $20,000 | 1.07 (0.95, 1.2) | 0.89 (0.68, 1.15) | 1.14 (0.67, 1.92) | 1.27 (0.82, 1.96) | 1.06 (0.68, 1.66) |
| < $25,000 | 1.02 (0.9, 1.14) | 1.03 (0.76, 1.39) | 0.82 (0.48, 1.41) | 1.27 (0.8, 2) | 1.06 (0.67, 1.66) |
| < $35,000 | 0.9 (0.79, 1.02) | 0.83 (0.61, 1.14) | 0.64 (0.35, 1.17) | 1.43 (0.83, 2.48) | 0.81 (0.49, 1.34) |
| < $50,000 | 0.79 (0.7, 0.89) | 0.74 (0.51, 1.08) | 0.61 (0.31, 1.19) | 1.48 (0.89, 2.46) | 0.96 (0.48, 1.93) |
| < $75,000 | 0.73 (0.64, 0.83) | 0.47 (0.3, 0.72) | 0.58 (0.32, 1.07) | 1.08 (0.6, 1.92) | 0.8 (0.46, 1.4) |
| $75,000 or higher | 0.58 (0.51, 0.66) | 0.63 (0.41, 0.96) | 0.45 (0.22, 0.94) | 1.49 (0.69, 3.22) | 0.31 (0.17, 0.54) |
| BMI | 1 (1, 1) | 1.01 (1, 1.02) | 1.01 (0.99, 1.04) | 1 (0.98, 1.02) | 1.03 (1.01, 1.04) |
| Employed | 0.72 (0.67, 0.77) | 0.72 (0.57, 0.9) | 0.66 (0.43, 1.01) | 0.73 (0.54, 0.99) | 0.63 (0.45, 0.87) |
| General Health Status |  | | | | |
| Excellent | 1.0 (ref) | | | | |
| Very Good | 1.52 (1.27, 1.83) | 1.13 (0.73, 1.76) | 0.84 (0.38, 1.84) | 1.64 (0.86, 3.15) | 1.72 (0.84, 3.5) |
| Good | 2.72 (2.28, 3.25) | 1.47 (0.99, 2.18) | 1 (0.49, 2.05) | 1.59 (0.97, 2.62) | 3.13 (1.55, 6.31) |
| Fair | 5.44 (4.54, 6.53) | 2.35 (1.57, 3.5) | 1.55 (0.71, 3.35) | 2.27 (1.28, 4) | 4.77 (2.37, 9.59) |
| Poor | 8.02 (6.62, 9.73) | 5.15 (3.33, 7.95) | 2.82 (1.2, 6.64) | 3.24 (1.84, 5.7) | 7.3 (3.39, 15.71) |
| Number of Days where Mental Health was not Good | 1.01 (1.01, 1.01) | 1.02 (1.01, 1.02) | 1.01 (1, 1.03) | 1.04 (1.02, 1.05) | 1.02 (1.01, 1.04) |
| Having Health Care Coverage | 1.08 (0.98, 1.2) | 1.34 (0.98, 1.83) | 1.47 (0.8, 2.7) | 1.46 (1.03, 2.07) | 1.23 (0.81, 1.86) |
| Access to Health Care Professionals |  | | | | |
| Yes, only one | 1.38 (1.25, 1.52) | 1 (0.77, 1.31) | 1.3 (0.84, 2) | 1.3 (0.91, 1.85) | 1.09 (0.75, 1.59) |
| More than one | 1.77 (1.56, 2.01) | 1.33 (0.96, 1.84) | 2.07 (1.18, 3.66) | 1.57 (1, 2.46) | 0.99 (0.64, 1.51) |
| No | 1.0 (ref) | | | | |
| Exercise in Past 30 Days | 0.83 (0.79, 0.88) | 0.86 (0.72, 1.03) | 1.05 (0.75, 1.46) | 0.76 (0.57, 1) | 0.91 (0.64, 1.29) |
| Alcohol in Past 30 Days | 0.86 (0.81, 0.92) | 0.84 (0.71, 1) | 0.92 (0.68, 1.25) | 0.97 (0.74, 1.27) | 0.94 (0.68, 1.29) |
| Length of Time Since Last Routine Checkup |  | | | | |
| Within Past Year | 1.36 (0.98, 1.88) | 1.37 (0.33, 5.69) | 1.99 (0.29, 13.72) | 0.54 (0.21, 1.35) | 0.94 (0.34, 2.61) |
| Within Past 2 Years | 1.18 (0.84, 1.66) | 1.12 (0.27, 4.72) | 1.43 (0.2, 10.25) | 0.51 (0.18, 1.43) | 1.05 (0.36, 3.06) |
| Within Past 5 Years | 1.12 (0.79, 1.59) | 1.19 (0.28, 5.09) | 1.29 (0.18, 9.47) | 0.91 (0.32, 2.57) | 1.01 (0.36, 2.88) |
| 5 or More Years Ago | 1.12 (0.79, 1.58) | 1 (0.23, 4.37) | 2.54 (0.35, 18.71) | 0.58 (0.22, 1.53) | 0.86 (0.3, 2.43) |
| Never | 1.0 (ref) | | | | |

Table S2. Multivariate Regression Analyses for Chronic Obstructive Pulmonary Disease among Former Smokers, BRFSS 2016-2018, 2020-21 Combined, Adjusted for Significantly Associated Covariates

|  | COPD |
| --- | --- |
|  | OR (95% CI) |
| Current E-Cigarette Use | 1.27 (1.13, 1.42) |
| Race | |
| NH-White | 1.0 (ref) |
| NH-Black | 0.67 (0.6, 0.74) |
| NH-AI/AN | 1.04 (0.88, 1.22) |
| Hispanic | 0.43 (0.38, 0.49) |
| NH-Other | 0.74 (0.62, 0.89) |
| Sex, Female | 1.17 (1.11, 1.23) |
| 18-24 | 1.0 (ref) |
| 25-34 | 0.92 (0.66, 1.26) |
| 35-44 | 1.05 (0.78, 1.42) |
| 45-54 | 1.68 (1.26, 2.24) |
| 55-64 | 2.7 (2.03, 3.59) |
| 65 and older | 3.22 (2.42, 4.28) |
| Region | |
| Northeast | 1.0 (ref) |
| Midwest | 1.03 (0.97, 1.1) |
| South | 1.08 (1.01, 1.16) |
| West | 0.97 (0.89, 1.05) |
| Education | |
| Less than high school | 1.0 (ref) |
| High school or more | 0.89 (0.83, 0.97) |
| Income | |
| < $10,000 | 1.0 (ref) |
| < $15,000 | 1.12 (0.99, 1.27) |
| < $20,000 | 1.07 (0.94, 1.21) |
| < $25,000 | 0.99 (0.88, 1.12) |
| < $35,000 | 0.92 (0.82, 1.03) |
| < $50,000 | 0.86 (0.76, 0.97) |
| < $75,000 | 0.77 (0.68, 0.88) |
| $75,000 or higher | 0.64 (0.56, 0.73) |
| BMI | 1.01 (1, 1.01) |
| Employed | 0.63 (0.59, 0.67) |
| Married | 0.84 (0.8, 0.89) |
| General Health Status | |
| Excellent | 1.0 (ref) |
| Very Good | 1.81 (1.58, 2.07) |
| Good | 3.39 (2.97, 3.87) |
| Fair | 6.33 (5.52, 7.25) |
| Poor | 10.53 (9.1, 12.18) |
| Number of Days where Mental Health was not Good | 1.01 (1.01, 1.01) |
| Access to Healthcare Professional | |
| Yes, only one | 1.23 (1.11, 1.36) |
| More than one | 1.46 (1.31, 1.64) |
| No | 1.0 (ref) |
| Smokeless Tobacco Use | |
| Every day | 0.84 (0.72, 0.97) |
| Some days | 0.9 (0.72, 1.12) |
| Not at all | 1.0 (ref) |
| Exercise in Past 30 Days | 0.77 (0.73, 0.81) |
| Alcohol in Past 30 Days | 0.87 (0.83, 0.92) |
| Length of Time Since Last Routine Checkup | |
| Within Past Year | 1.36 (0.94, 1.97) |
| Within Past 2 Years | 1.07 (0.74, 1.57) |
| Within Past 5 Years | 1.19 (0.8, 1.77) |
| 5 or More Years Ago | 1 (0.67, 1.49) |
| Never | 1.0 (ref) |

Table S3. Multivariate Weighted Regression Analyses for Chronic Obstructive Pulmonary Disease among Never Smokers, Stratified by Race, BRFSS 2016-2018, 2020-21 Combined, Adjusted for Significantly Associated Covariates

|  | COPD | | | | |
| --- | --- | --- | --- | --- | --- |
|  | NH-White | NH-Black | NH-AI/AN | Hispanic | NH-Other |
|  | OR (95% CI) | | | | |
| Current E-Cigarette Use | 1.49 (1.13, 1.95) | 2.01 (1.07, 3.78) | 3.23 (0.53, 19.83) | 1.76 (1.08, 2.87) | 0.66 (0.29, 1.52) |
| Sex, Female | 1.26 (1.18, 1.35) | 1.22 (1.02, 1.45) | 1.75 (1.21, 2.54) | 1.19 (0.99, 1.44) | 0.98 (0.72, 1.34) |
| Age Group |  | | | | |
| 18-24 | 1.0 (ref) | | | | |
| 25-34 | 1.12 (0.91, 1.37) | 1.3 (0.91, 1.88) | 3.39 (1.23, 9.31) | 1.71 (1.15, 2.52) | 1.87 (1.04, 3.37) |
| 35-44 | 1.32 (1.07, 1.62) | 1.3 (0.89, 1.9) | 4.07 (1.3, 12.76) | 2.06 (1.4, 3.03) | 2.14 (1.16, 3.97) |
| 45-54 | 1.89 (1.57, 2.27) | 1.68 (1.18, 2.38) | 5.49 (2.14, 14.07) | 2.03 (1.4, 2.95) | 3.03 (1.64, 5.58) |
| 55-64 | 2.55 (2.15, 3.04) | 2.04 (1.44, 2.88) | 6.38 (2.53, 16.07) | 1.97 (1.33, 2.91) | 4.19 (2.28, 7.7) |
| 65 and older | 3.09 (2.6, 3.66) | 2.07 (1.49, 2.86) | 6.19 (2.51, 15.29) | 2.87 (1.94, 4.26) | 4.89 (2.66, 9) |
| Region |  | | | | |
| Northeast | 1.0 (ref) | | | | |
| Midwest | 1.03 (0.94, 1.13) | 1.31 (1.04, 1.66) | 0.79 (0.41, 1.52) | 0.92 (0.67, 1.26) | 1.01 (0.69, 1.47) |
| South | 1.19 (1.09, 1.3) | 1.13 (0.91, 1.4) | 1.28 (0.7, 2.34) | 0.98 (0.79, 1.22) | 1.26 (0.83, 1.9) |
| West | 1.16 (1.04, 1.29) | 1.1 (0.76, 1.59) | 0.52 (0.28, 0.97) | 1.13 (0.9, 1.41) | 0.77 (0.52, 1.15) |
| Income |  | | | | |
| < $10,000 | 1.0 (ref) | | | | |
| < $15,000 | 0.99 (0.83, 1.19) | 0.98 (0.73, 1.33) | 1.93 (0.97, 3.85) | 1.1 (0.74, 1.64) | 0.89 (0.42, 1.9) |
| < $20,000 | 1 (0.83, 1.19) | 1.01 (0.77, 1.33) | 1.19 (0.57, 2.45) | 0.81 (0.58, 1.13) | 0.76 (0.38, 1.53) |
| < $25,000 | 0.85 (0.71, 1.01) | 0.88 (0.67, 1.16) | 1 (0.49, 2.03) | 0.76 (0.53, 1.09) | 0.55 (0.29, 1.05) |
| < $35,000 | 0.78 (0.65, 0.92) | 0.73 (0.54, 1) | 1.86 (0.9, 3.81) | 0.75 (0.53, 1.07) | 0.45 (0.24, 0.86) |
| < $50,000 | 0.73 (0.61, 0.86) | 0.62 (0.46, 0.85) | 1.28 (0.6, 2.77) | 0.86 (0.56, 1.3) | 0.45 (0.24, 0.85) |
| < $75,000 | 0.64 (0.54, 0.77) | 0.64 (0.45, 0.91) | 0.54 (0.24, 1.22) | 0.8 (0.51, 1.25) | 0.52 (0.26, 1.03) |
| $75,000 or higher | 0.54 (0.45, 0.64) | 0.61 (0.44, 0.83) | 1 (0.47, 2.13) | 0.79 (0.54, 1.16) | 0.45 (0.24, 0.87) |
| BMI | 1.03 (1.02, 1.03) | 1.01 (1, 1.02) | 1.01 (0.99, 1.03) | 1.01 (1, 1.03) | 1.06 (1.04, 1.08) |
| Employed | 0.73 (0.67, 0.79) | 0.73 (0.6, 0.89) | 0.48 (0.28, 0.83) | 0.9 (0.73, 1.12) | 0.86 (0.58, 1.29) |
| Number of Children in the Household | 0.99 (0.95, 1.04) | 1.02 (0.97, 1.08) | 0.93 (0.78, 1.1) | 0.86 (0.78, 0.94) | 0.9 (0.79, 1.02) |
| Married | 0.83 (0.77, 0.9) | 0.83 (0.69, 1) | 1.31 (0.85, 2) | 0.82 (0.66, 1.01) | 0.81 (0.58, 1.14) |
| General Health Status |  | | | | |
| Excellent | 1.0 (ref) | | | | |
| Very Good | 1.58 (1.37, 1.82) | 1.21 (0.87, 1.69) | 1.26 (0.52, 3.05) | 1.21 (0.8, 1.81) | 1.69 (0.81, 3.5) |
| Good | 3.04 (2.63, 3.5) | 1.77 (1.31, 2.39) | 1.27 (0.57, 2.82) | 1.53 (1.04, 2.25) | 1.66 (0.84, 3.28) |
| Fair | 5.67 (4.87, 6.6) | 3.48 (2.6, 4.65) | 3.19 (1.38, 7.37) | 3.41 (2.3, 5.05) | 4.09 (1.9, 8.77) |
| Poor | 9.51 (8, 11.3) | 5.22 (3.72, 7.35) | 6.39 (2.62, 15.59) | 4.71 (3.02, 7.33) | 7.9 (3.37, 18.52) |
| Number of Days where Mental Health was not Good | 1.02 (1.01, 1.02) | 1.02 (1.01, 1.03) | 1.02 (1, 1.04) | 1.03 (1.02, 1.04) | 1.01 (0.99, 1.03) |
| Access to Health Care Professionals |  | | | | |
| Yes, only one | 1.1 (0.97, 1.24) | 1 (0.78, 1.29) | 2.14 (1.26, 3.64) | 1.19 (0.92, 1.54) | 1.19 (0.81, 1.73) |
| More than one | 1.31 (1.14, 1.5) | 1.55 (1.15, 2.08) | 3.17 (1.65, 6.11) | 1.88 (1.35, 2.64) | 1.51 (0.91, 2.51) |
| No | 1.0 (ref) | | | | |
| Exercise in Past 30 Days | 0.88 (0.82, 0.94) | 0.8 (0.69, 0.94) | 0.7 (0.49, 1) | 1.21 (1, 1.46) | 1.06 (0.78, 1.45) |

Table S4. Multivariate Weighted Regression Analyses for Asthma Status (Current vs. Never) among Current Smokers, Stratified by Race, BRFSS 2016-2018, 2020-21 Combined, Adjusted for Significantly Associated Covariates

|  | Asthma | | | | |
| --- | --- | --- | --- | --- | --- |
|  | Current vs. Never | | | | |
|  | NH-White | NH-Black | NH-AI/AN | Hispanic | NH-Other |
|  | OR (95% CI) | | | | |
| Current E-Cigarette Use | 1.2 (1.09, 1.31) | 1.48 (1.14, 1.93) | 0.96 (0.6, 1.54) | 1.97 (1.45, 2.67) | 1.5 (1.03, 2.18) |
| Sex, Female | 1.82 (1.7, 1.95) | 1.65 (1.39, 1.96) | 1.71 (1.22, 2.41) | 2.69 (2.12, 3.41) | 1.88 (1.44, 2.46) |
| Age Group |  | | | | |
| 18-24 | 1.0 (ref) | | | | |
| 25-34 | 0.78 (0.66, 0.92) | 1.44 (0.89, 2.32) | 0.65 (0.28, 1.51) | 1.18 (0.81, 1.72) | 1.11 (0.66, 1.84) |
| 35-44 | 0.71 (0.6, 0.84) | 1.15 (0.71, 1.86) | 0.61 (0.27, 1.38) | 0.76 (0.51, 1.12) | 0.94 (0.54, 1.63) |
| 45-54 | 0.61 (0.52, 0.72) | 0.94 (0.58, 1.52) | 0.74 (0.34, 1.6) | 0.8 (0.51, 1.25) | 0.89 (0.51, 1.55) |
| 55-64 | 0.5 (0.43, 0.59) | 0.82 (0.5, 1.33) | 0.45 (0.2, 1.05) | 0.55 (0.36, 0.85) | 0.89 (0.53, 1.51) |
| 65 and older | 0.36 (0.3, 0.43) | 0.47 (0.28, 0.78) | 0.42 (0.17, 1.04) | 0.57 (0.33, 1) | 0.65 (0.34, 1.22) |
| Region |  | | | | |
| Northeast | 1.0 (ref) | | | | |
| Midwest | 0.81 (0.74, 0.89) | 0.96 (0.76, 1.21) | 0.56 (0.31, 1.01) | 0.43 (0.31, 0.58) | 1.12 (0.77, 1.64) |
| South | 0.81 (0.74, 0.89) | 0.66 (0.53, 0.81) | 0.68 (0.38, 1.22) | 0.4 (0.31, 0.53) | 1.04 (0.71, 1.53) |
| West | 0.98 (0.88, 1.1) | 0.97 (0.67, 1.42) | 0.52 (0.29, 0.94) | 0.47 (0.36, 0.62) | 1.06 (0.73, 1.54) |
| Education |  | | | | |
| Less than high school | 1.0 (ref) | | | | |
| High school or more | 0.73 (0.67, 0.8) | 0.64 (0.53, 0.78) | 1.06 (0.73, 1.54) | 1.06 (0.84, 1.34) | 0.67 (0.49, 0.91) |
| Income |  | | | | |
| < $10,000 | 1.0 (ref) | | | | |
| < $15,000 | 0.97 (0.84, 1.11) | 0.86 (0.65, 1.13) | 1.27 (0.77, 2.1) | 0.67 (0.47, 0.96) | 1.07 (0.68, 1.68) |
| < $20,000 | 0.89 (0.78, 1.01) | 0.86 (0.66, 1.12) | 1.61 (0.98, 2.66) | 1.01 (0.7, 1.45) | 0.78 (0.48, 1.24) |
| < $25,000 | 0.78 (0.69, 0.89) | 0.8 (0.59, 1.08) | 1.05 (0.62, 1.79) | 0.82 (0.55, 1.21) | 0.61 (0.38, 0.96) |
| < $35,000 | 0.68 (0.59, 0.77) | 0.84 (0.63, 1.11) | 1.16 (0.58, 2.32) | 1.28 (0.84, 1.94) | 0.79 (0.47, 1.33) |
| < $50,000 | 0.57 (0.49, 0.66) | 0.85 (0.6, 1.2) | 0.96 (0.5, 1.86) | 0.95 (0.63, 1.43) | 0.58 (0.37, 0.91) |
| < $75,000 | 0.62 (0.52, 0.73) | 0.62 (0.42, 0.91) | 0.56 (0.29, 1.07) | 0.98 (0.61, 1.59) | 0.5 (0.29, 0.84) |
| $75,000 or higher | 0.55 (0.47, 0.64) | 0.68 (0.47, 0.98) | 0.52 (0.26, 1.05) | 0.89 (0.54, 1.45) | 0.8 (0.47, 1.35) |
| BMI | 1.02 (1.02, 1.03) | 1.02 (1.01, 1.03) | 1 (0.98, 1.02) | 1.02 (1.01, 1.04) | 1.04 (1.02, 1.06) |
| Employed | 0.88 (0.81, 0.95) | 0.77 (0.64, 0.93) | 0.7 (0.45, 1.1) | 0.66 (0.52, 0.85) | 0.77 (0.57, 1.04) |
| Married | 0.93 (0.87, 1) | 0.95 (0.78, 1.17) | 0.69 (0.49, 0.98) | 0.9 (0.69, 1.17) | 0.84 (0.62, 1.14) |
| General Health Status |  | | | | |
| Excellent | 1.0 (ref) | | | | |
| Very Good | 1.3 (1.12, 1.52) | 1.11 (0.79, 1.58) | 2.28 (1.03, 5.06) | 1.38 (0.93, 2.04) | 1.29 (0.61, 2.72) |
| Good | 2.02 (1.74, 2.34) | 1.38 (0.99, 1.91) | 1.73 (0.84, 3.54) | 1.51 (1.04, 2.2) | 1.51 (0.74, 3.09) |
| Fair | 3.43 (2.94, 4.01) | 1.91 (1.37, 2.67) | 3.48 (1.67, 7.25) | 2.15 (1.46, 3.17) | 2.03 (0.98, 4.22) |
| Poor | 4.62 (3.89, 5.49) | 3.28 (2.24, 4.81) | 7.44 (3.29, 16.83) | 4.19 (2.64, 6.64) | 2.86 (1.32, 6.22) |
| Number of Days where Mental Health was not Good | 1.01 (1.01, 1.02) | 1.01 (1.01, 1.02) | 1.01 (1, 1.03) | 1.04 (1.03, 1.05) | 1.04 (1.02, 1.05) |
| Access to Health Care Professionals |  | | | | |
| Yes, only one | 1.37 (1.24, 1.51) | 1.44 (1.13, 1.83) | 0.96 (0.64, 1.45) | 1.6 (1.21, 2.1) | 1.3 (0.93, 1.8) |
| More than one | 1.59 (1.39, 1.81) | 1.55 (1.15, 2.09) | 1.38 (0.81, 2.35) | 1.88 (1.3, 2.71) | 1.05 (0.69, 1.59) |
| No | 1.0 (ref) | | | | |
| Exercise in Past 30 Days | 0.99 (0.92, 1.06) | 1.01 (0.85, 1.19) | 1.24 (0.87, 1.75) | 0.99 (0.79, 1.24) | 0.85 (0.65, 1.11) |
| Alcohol in Past 30 Days | 0.87 (0.81, 0.93) | 0.99 (0.84, 1.17) | 0.79 (0.57, 1.1) | 0.76 (0.61, 0.96) | 0.74 (0.57, 0.96) |
| Length of Time Since Last Routine Checkup |  | | | | |
| Within Past Year | 1.76 (1.03, 3.04) | 3.65 (1.15, 11.56) | 4.15 (0.87, 19.85) | 2.26 (0.8, 6.37) | 2.93 (0.79, 10.9) |
| Within Past 2 Years | 1.64 (0.95, 2.84) | 3.09 (0.96, 9.94) | 3.31 (0.63, 17.38) | 2.31 (0.81, 6.56) | 3.4 (0.9, 12.87) |
| Within Past 5 Years | 1.33 (0.77, 2.29) | 2.54 (0.78, 8.28) | 2.4 (0.49, 11.73) | 1.74 (0.61, 5) | 1.83 (0.46, 7.28) |
| 5 or More Years Ago | 1.25 (0.73, 2.16) | 2.36 (0.71, 7.82) | 2.31 (0.45, 11.92) | 3.14 (1.08, 9.17) | 2.34 (0.61, 8.95) |
| Never | 1.0 (ref) | | | | |

Table S5. Multivariate Weighted Regression Analyses for Asthma Status among Former and Never Smokers, BRFSS 2016-2018, 2020-21 Combined, Adjusted for Significantly Associated Covariates

|  | Asthma (current vs. never) | |
| --- | --- | --- |
|  | OR (95% CI) | |
| Smoking Status | Former | Never |
| Current E-Cigarette Use | 0.95 (0.86, 1.05) | 1.17 (1.02, 1.34) |
| Race |  | |
| NH-White | 1.0 (ref) | |
| NH-Black | 1.06 (0.97, 1.17) | 1.03 (0.97, 1.09) |
| NH-AI/AN | 1.34 (1.13, 1.58) | 1.17 (1.02, 1.35) |
| Hispanic | 0.79 (0.71, 0.88) | 0.71 (0.66, 0.76) |
| NH-Other | 1.05 (0.92, 1.19) | 0.69 (0.64, 0.76) |
| Age Groups |  | |
| 18-24 | 1.0 (ref) | |
| 25-34 | 0.69 (0.57, 0.83) | 0.83 (0.77, 0.89) |
| 35-44 | 0.66 (0.55, 0.79) | 0.71 (0.65, 0.76) |
| 45-54 | 0.58 (0.48, 0.69) | 0.69 (0.64, 0.74) |
| 55-64 | 0.56 (0.47, 0.67) | 0.62 (0.58, 0.67) |
| 65 and older | 0.47 (0.39, 0.56) | 0.5 (0.46, 0.54) |
| Sex, Female | 1.96 (1.86, 2.05) | 1.70 (1.64, 1.77) |
| Region |  | |
| Northeast | 1.0 (ref) | |
| Midwest | 0.91 (0.85, 0.96) | 0.851 (0.812, 0.892) |
| South | 0.83 (0.78, 0.88) | 0.841 (0.802, 0.882) |
| West | 1.1 (1.02, 1.18) | 1.018 (0.964, 1.075) |
| Education |  | |
| Less than high school | 1.0 (ref) | |
| High school and up | -- | 1.22 (1.12, 1.34) |
| Income |  | |
| < $10,000 | 1.0 (ref) | |
| < $15,000 | 0.95 (0.83, 1.09) | 0.89 (0.79, 1.00) |
| < $20,000 | 0.86 (0.75, 0.98) | 0.9 (0.81, 1.00) |
| < $25,000 | 0.88 (0.77, 1) | 0.83 (0.75, 0.92) |
| < $35,000 | 0.77 (0.68, 0.87) | 0.76 (0.69, 0.84) |
| < $50,000 | 0.7 (0.62, 0.79) | 0.78 (0.70, 0.86) |
| < $75,000 | 0.71 (0.63, 0.81) | 0.83 (0.75, 0.92) |
| $75,000 or higher | 0.75 (0.66, 0.86) | 0.89 (0.80, 0.98) |
| BMI | 1.03 (1.03, 1.03) | 1.03 (1.03, 1.04) |
| Number of Children | -- | 0.97 (0.96, 0.99) |
| Employed | 0.86 (0.81, 0.91) | 0.87 (0.83, 0.91) |
| Married | 0.94 (0.89, 0.99) | 0.87 (0.83, 0.91) |
| General Health Status |  | |
| Excellent | 1.0 (ref) | |
| Very Good | 1.48 (1.35, 1.63) | 1.47 (1.39, 1.56) |
| Good | 2.09 (1.9, 2.3) | 1.90 (1.79, 2.02) |
| Fair | 3.14 (2.82, 3.48) | 2.84 (2.64, 3.06) |
| Poor | 4.31 (3.82, 4.87) | 3.89 (3.51, 4.31) |
| Having Health Care Coverage | -- | 1.27 (1.168, 1.39) |
| Number of Days where Mental Health was not Good | 1.02 (1.01, 1.02) | 1.02 (1.02, 1.02) |
| Access to Health Care Professionals |  | |
| Yes, only one | 1.46 (1.33, 1.62) | 1.39 (1.30, 1.48) |
| More than one | 1.57 (1.4, 1.76) | 1.55 (1.44, 1.67) |
| No | 1.0 (ref) | |
| Exercise in Past 30 Days | -- | 1.02 (0.98, 1.07) |
| Alcohol in Past 30 Days | -- | 1.06 (1.02, 1.10) |
| Length of Time Since Last Routine Checkup |  | |
| Within Past Year | 1.37 (0.87, 2.15) | 1.49 (1.14, 1.97) |
| Within Past 2 Years | 1.21 (0.77, 1.91) | 1.28 (0.97, 1.69) |
| Within Past 5 Years | 1.13 (0.71, 1.8) | 1.29 (0.97, 1.71) |
| 5 or More Years Ago | 0.99 (0.62, 1.59) | 1.09 (0.82, 1.45) |
| Never | 1.0 (ref) | |
